# Drug-inducible synergistic gene silencing with multiple small hairpin RNA molecules for gene function study in animal model

**DOI:** 10.1007/s11248-014-9841-9

**Published:** 2014-10-01

**Authors:** Ming Ying, Guangfeng Chen, Yu Qiu, Xiujuan Shi, Chen Zhang, Qiuke Wang, Shuzhang Yang, Lixia Lu, Qionglan Yuan, Guotong Xu, Zibing Jin, Qiang Wu, Xiaoqing Liu

**Affiliations:** 1 Shanghai Tenth People’s Hospital, Tongji University School of Medicine, 301 Yanchangzhong Rd., Shanghai, 200072 China; 2 Shenzhen Key Laboratory of Marine Bioresources and Ecology, College of Life Sciences, Shenzhen University, 3688 Nanhai Ave., Shenzhen, 518060 China; 3 Division of Ophthalmic Genetics, The Eye Hospital of Wenzhou Medical College and Lab for Stem Cell and Retinal Regeneration, School of Ophthalmology and Optometry, Wenzhou Medical College, No. 270, West Xueyuan Rd., Wenzhou, 325027 China; 4 Department of Ophthalmology, Affiliated Sixth People’s Hospital, Shanghai Jiao Tong University, No. 600 Yishan Rd., Shanghai, 200233 China

**Keywords:** RNAi, shRNA, Gene targeting, Gene silencing, Drug-inducible

## Abstract

Gene targeting is a critical tool for construction of disease models. However, the application of traditional homologous recombination-mediated gene knockout technology is limited by the absence of rapid frequency-guaranteed targeting methods. Although conventional small hairpin RNA (shRNA)-mediated gene silencing offers an alternative for gene targeting, its application is frequently compromised by lower expression efficiency via RNA interference compared to gene knockout. Here we provide an efficient gene targeting strategy involving drug-inducible synergistic silencing with multiple shRNA molecules. On induction, the levels of the target proteins decreased to undetectable levels in all the tested stable transgenic mammalian cell lines, including HEK293 and embryonic stem cell-derived progenies carrying shRNA silencing cassettes. In a transgenic mouse model carrying a silencing cassette targeting the rhodopsin gene, short-time inducer treatment was sufficient to ablate the rhodopsin protein in the retina, resulting in similar retinal phenotypic changes as those observed in rhodopsin mutant mice. Therefore, on a broad basis, this inducible shRNA gene targeting strategy offers a true gene knockout alternative comparable to conventional RNA interference approaches.

**Electronic supplementary material:**

The online version of this article (doi:10.1007/s11248-014-9841-9) contains supplementary material, which is available to authorized users.

## Introduction

Gene targeting is a key tool for construction of disease models, and it plays a critical role in diverse fields of biological and medical studies, including gene function analysis, gene modification, and drug development. Of note, traditional homologous recombination-mediated gene knockout technology is limited by the absence of rapid frequency-guaranteed targeting methods (Santiago et al. 2008). Recently, zinc-finger nuclease-mediated gene editing or transcription activator-like effector nucleases (TALEN) and Cas9 RNA-guide endonuclease technology appears to be receiving increasing attention for gene manipulation; however, targeted disruption of lethal genes remains an unsolved problem (Cho et al. 2013; Gaj et al. 2012, 2013; Sung et al. 2013; Urnov et al. 2005). The field of developmental biology has greatly benefited from the use of an evolving set of genetic tools. Utilizing conditional, recombination-based strategies, genes can be deleted in a specific cell type and in some instances with temporal control. Although conditional gene inactivation theoretically appears to be an excellent strategy for gene deletion in specific cell types, the maintenance of animal models involves much more labor than that of straight gene knockout models. Although the utilization of interference (RNAi) for targeted gene silencing in mammalian cells has become a benchmark technology, related strategies remain to be developed for higher gene silencing efficiency (Lambeth and Smith 2013). To modify gene inactivation methods, we propose an efficient gene strategy that involves drug-inducible synergistic silencing with multiple small hairpin RNA (shRNA) molecules.

## Materials and methods

### Constructs

ShRNA silencing vectors were generated using standard cloning procedures. Plasmid DNA was prepared using a QIAprep Spin Miniprep Kit (Qiagen). We constructed the backbone vector pPolyshRNA by replacing the Tet-U6-MCS region of the pSingle-MCS-shRNA vector (Clontech Laboratories, Inc.) with an shRNA silencing cassette (P_DRE1_–*Xho*I–shRNA1–*Age*I–P_DRE2_–*Sal*I–shRNA2–*Mlu*I–P_DRE3_–*Pst*I–shRNA3–*Hin*dIII), allowing three shRNA subcassettes to be synergistically transcribed under tight control of doxycycline-responsive promoters (P_DRE1_, P_DRE2_, and P_DRE3_). Oligonucleotides matching the sequences of the designed shRNAs were synthesized, annealed, and sequentially subcloned into the pPolyshRNA vector. The sequences of P_DRE1_, P_DRE2_, and P_DRE3_ and the oligonucleotides matching the designed shRNA sequences targeting the genes of interest are listed in supplementary materials (Supplementary Tables S1–6).

### Cell culture

HEK293 cells and mouse embryonic stem (ES) cells (129/Sv line) were used to generate stable transgenic cell lines. HEK293 cells were cultured in Dulbecco’sModified Eagle Medium (DMEM) supplemented with 10 % fetal bovine serum (FBS). To generate stable transgenic cell lines targeting the SIRT1 gene, HEK293 cells were transfected with shRNA vectors targeting the SIRT1 gene. Stable transgenic cell lines were selected by supplementing with 400 µg/ml G418. ES cells were cultivated on irradiated mouse embryonic feeder (MEFs) in DMEM containing 15 % FBS, leukemia-inhibiting factor (LIF), penicillin/streptomycin, l-glutamine, and non-essential amino acids. Trypsinization (0.25 % trypsin, 1 mM EDTA, 37 °C, 3–5 min) was used to dissociate cell clumps into single cells; these were then transduced with a 40-µg DNA vector by electroporation (250 V, 500 µF). After 48 h, G418 (400 µg/ml) was added to select stable transgenic cells, and culture was continued for 7–10 days. qRT-PCR, immunoblotting, and immunofluorescence staining were performed after at least 7–10 days of doxycycline treatment. Stepwise-differentiation of mouse ES cells into photoreceptor cells was performed as previously described (Osakada et al. 2008). Gene expression was analyzed by qRT-PCR, immunoblotting, and immunofluorescence staining after 7-day doxycycline treatment.

### Animals

Wild-type (WT) mice carrying a mixed genetic background of C57BL/6 and 129/Sv strains were used as positive controls. ES cell clones integrated with shRNA silencing alleles were used to microinject C57BL/6 blastocysts. The resultant chimeras were crossed with CB57BL/6 to generate heterozygotes. Genotyping was performed using genomic PCR. Primers (N5: 5′-gctgctctgatgccgccgtgttc; N3: 5′-gatgtttcgcttggtggtcgaatg) matching a partial sequence of the neomycin-resistance gene were used to amplify transgenic alleles. Newborn mice were intraperitoneally administered with or without 2 µg doxycycline per gram body weight every day until they were sacrificed (about 2 months). qRT-PCR, immunoblotting, and immunofluorescence staining were used to evaluate silencing efficiency after doxycycline treatment. Mouse eyes were enucleated and eye cups were subjected to morphological analysis. Retinas were subjected to protein analysis. All experiments involving animals were performed in accordance with the National Institutes of Health Guide for the Care and Use of Laboratory Animals and approved by the Biological Research Ethics Committee, Chinese Academy of Sciences.

### Quantitative real-time PCR

Total RNA was isolated and reverse transcription was performed as described, and quantitative real-time PCR (qRT-PCR) was performed to evaluate the mRNA levels of genes of interest (Chen et al. 2013). qRT-PCR was performed using Platinum SYBR Green qPCR SuperMixUDG with Rox (Invitrogen). mRNA levels of actin were determined as internal controls. Corresponding oligonucleotides for determining the mRNA levels of SIRT1, rhodopsin and actin are provided in supplementary materials (Supplementary Table S7).

### Immunofluorescence analysis and immunoblotting

Immunoblotting and immunofluorescence staining were performed to determine the levels and distributions of target proteins. Frozen sections were cut after fixation with 4 % formaldehyde at room temperature for 10 min. Paraffin sections were prepared for hematoxylin and eosin (HE) staining to evaluate the morphological changes in transgenic mouse retinas. The antibodies and working dilutions used for immunofluorescence analysis and immunoblotting were as follows: rabbit anti-SIRT1 (1:500–2,000; #1104-1, Epitomics), anti-rhodopsin (1:500–2,000; #sc-57433, Santa Cruz), rabbit anti-GNAT1 (1:200–1,000; # sc-389, Santa Cruz), goat anti-CNGA1 (1:500–1,000; #sc-13694, Santa Cruz), goat anti-PDC (1:500–1,000; #sc-18413, Santa Cruz), anti-PDE6b (1:200–1,000; #sc-30717, Santa Cruz), anti-actin (1:2,000–10,000; #A5228, Sigma-Aldrich). Corresponding IgG antibodies conjugated with Alexa Fluor^®^ dye (594 or 488; Invitrogen) were used as secondary antibodies for the immunofluorescence analysis. In immunoblotting, anti-mouse IgG peroxidase conjugate (1:50,000; #a2304, Sigma-Aldrich), goat anti-rabbit IgG peroxidase conjugate (1:50,000; #a9169, Sigma-Aldrich), and rabbit anti-goat IgG peroxidase conjugate (1:80,000; #a5420, Sigma-Aldrich) were used as probes for the proteins of interest.

## Result

### Targeting scheme

**Fig. 1:**
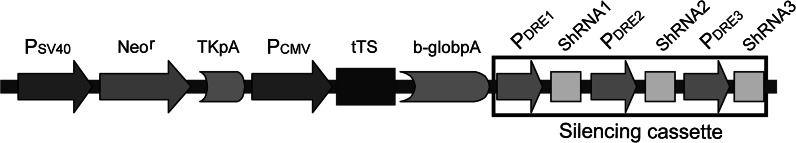
Gene silencing design: Element arrangement for the silencing vector pPolyshRNA. The three shRNA silencing subcassettes are *boxed* as indicated. Given that the vector carries a neomycin-resistance allele, stable cell lines that integrated the three silencing cassettes could be selected by G418. The tTS regulator is constitutively expressed under the control of the CMV promoter. *tTS* tetracycline-controlled transcriptional silencer, *CMV* cytomegalovirus

Tetracycline-responsible elements (TREs) or their modified doxycycline-responsive elements (DREs) have been widely applied in inducible expression systems (Bockamp et al. 2007; Freundlieb et al. 1999; Henriksen et al. 2007; Lai et al. 2004; Lamartina et al. 2003; Zhu et al. 2002). Tetracycline-controlled transcriptional silencer (tTS), a fusion protein composed of the tet repressor and the KRAB-AB domain of the kid-1 transcriptional repressor, is known to tightly bind with the RNA polymerase-binding site in the absence of doxycycline, resulting in very low basal expression of directed silencing subcassettes (Zhu et al. 2001). To develop an efficient inducible silencing system, we utilized the tTS silencer in this study, whose expression was constitutively driven by the CMV promoter. We also aligned multiple silencing subcassettes in a single vector with each subcassette controlled by its own independent P_DRE_. To minimize the potential reciprocal interference of the RNA polymerase-binding domains, we designed the promoters (P_DRE1_, P_DRE2_, and P_DRE3_) by hybridizing a doxycycline-responsive element and U6 promoter with a spacer (100–200 nucleotides) inserted between independent silencing subcassettes, resulting in a backbone vector (pPolyshRNA) (Fig. 1). Thus, this vector allowed multiple shRNA molecules to be synergistically transcribed on induction by doxycycline.

### Gene silencing in mammalian cell lines

**Fig. 2:**
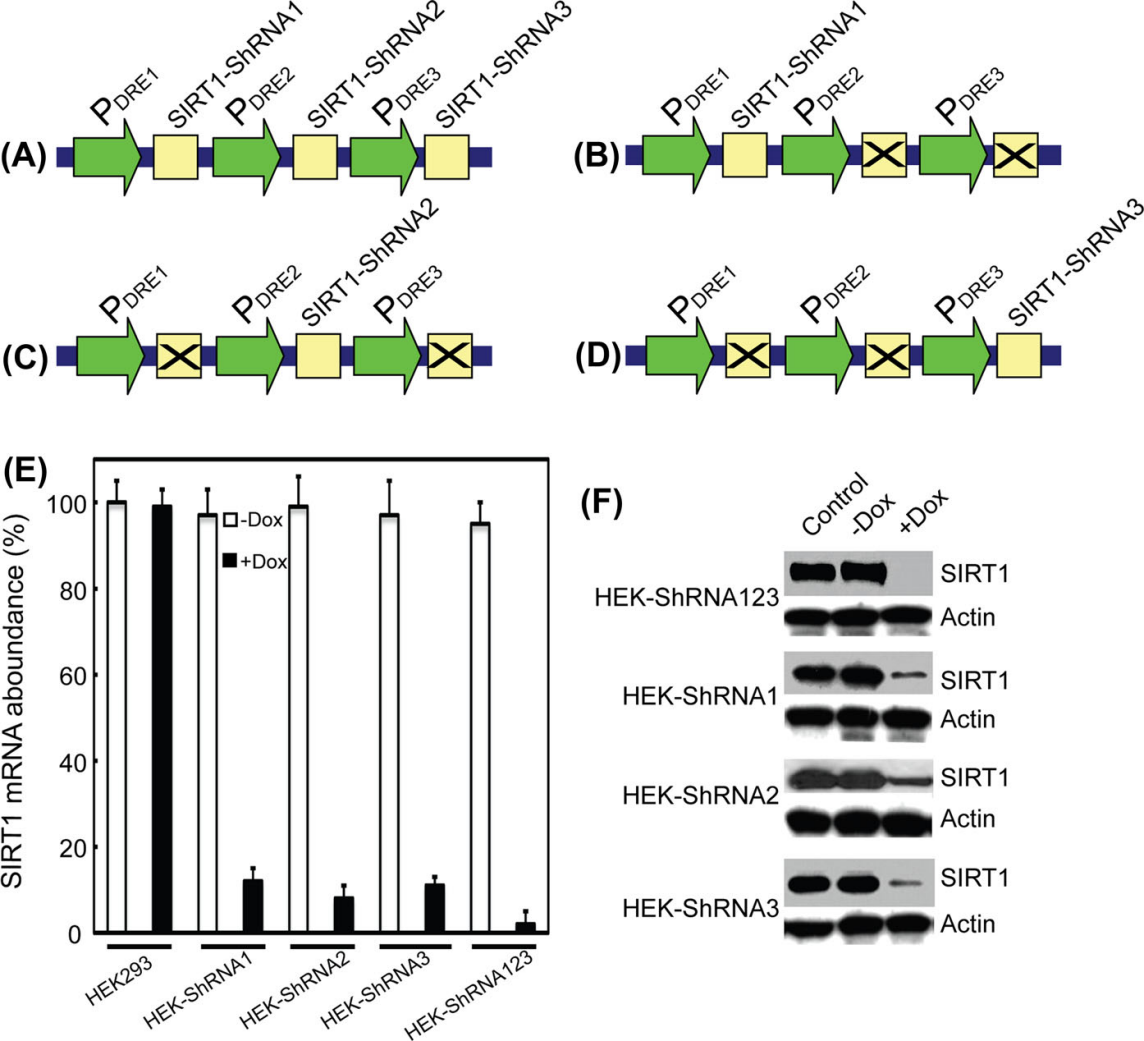
Silencing of SIRT1 expression in HEK293 cells. **a** Targeting schemes for SIRT1 gene silencing with three shRNAs (SIRT1–shRNA1, SIRT1–shRNA2, and SIRT1–shRNA3). **b-d** Targeting schemes for the vectors harboring individual shRNA subcassettes (SIRT1–shRNA1, SIRT1–shRNA2, or SIRT1–shRNA3). **e** SIRT1 mRNA levels. **f** SIRT1 protein levels. HEK–shRNA123, HEK–shRNA1, HEK–shRNA2, and HEK–shRNA3, were integrated with the DNA vectors harboring the silencing subcassettes as indicated in **a**–**d**, respectively. Cells were analyzed after 1-week doxycycline treatment. Normal HEK293 cells were included as control. Values are mean ± SEM (n = 3, *P* < 0.01)

To test the silencing efficiency of this strategy, we targeted the human SIRT1 gene (GenBank accession number, NM_012238.4) with three different shRNA subcassettes (SIRT1–shRNA1, SIRT1–shRNA2, and SIRT1–shRNA3) assembled in the pPolyshRNA vector (Fig. 2a) (Supplementary Table S2). To examine the silencing efficiency of each shRNA, we also constructed vectors for each individual shRNA in the absence of the other two shRNA molecules (Fig. 2b–d). qRT-PCR revealed that the gene silencing efficiency ranged from 88 to 93 % for each individual shRNA subcassette in stable transgenic HEK293 cell lines after doxycycline treatment. More strikingly, the silencing efficiency was up to 98 % with all the three shRNA subcassettes placed in a single vector, indicative of the obvious additive effect of these shRNA subcassettes (Fig. 2e). Immunobotting showed that 10–30 % SIRT1 was expressed in the presence of any one of these shRNA subcassettes alone, whereas it was below the detectable level in the presence of all three shRNA subcassettes in a single vector (Fig. 2f), indicating that the residual traceable amount of SIRT1 mRNA might not serve as an effective template for SIRT1 protein production. These observations suggested that the shRNA molecules probably not only targeted SIRT1 mRNA to degradative pathways but also blocked the translation process by binding to the residual SIRT1 mRNA molecules.

Furthermore, we examined the silencing efficiency of each individual shRNA subcassette by increasing its copy number in a single vector. We constructed three vectors by placing each identical shRNA subcassette under the control of all the silencing subcassettes in the pPolyshRNA vector (Fig. S1a-c). qRT-PCR and immunoblotting showed that silencing efficiency was about 90 % (Fig. S1d,e).

### Gene silencing in photoreceptor cells differentiated from embryonic stem cells

**Fig. 3:**
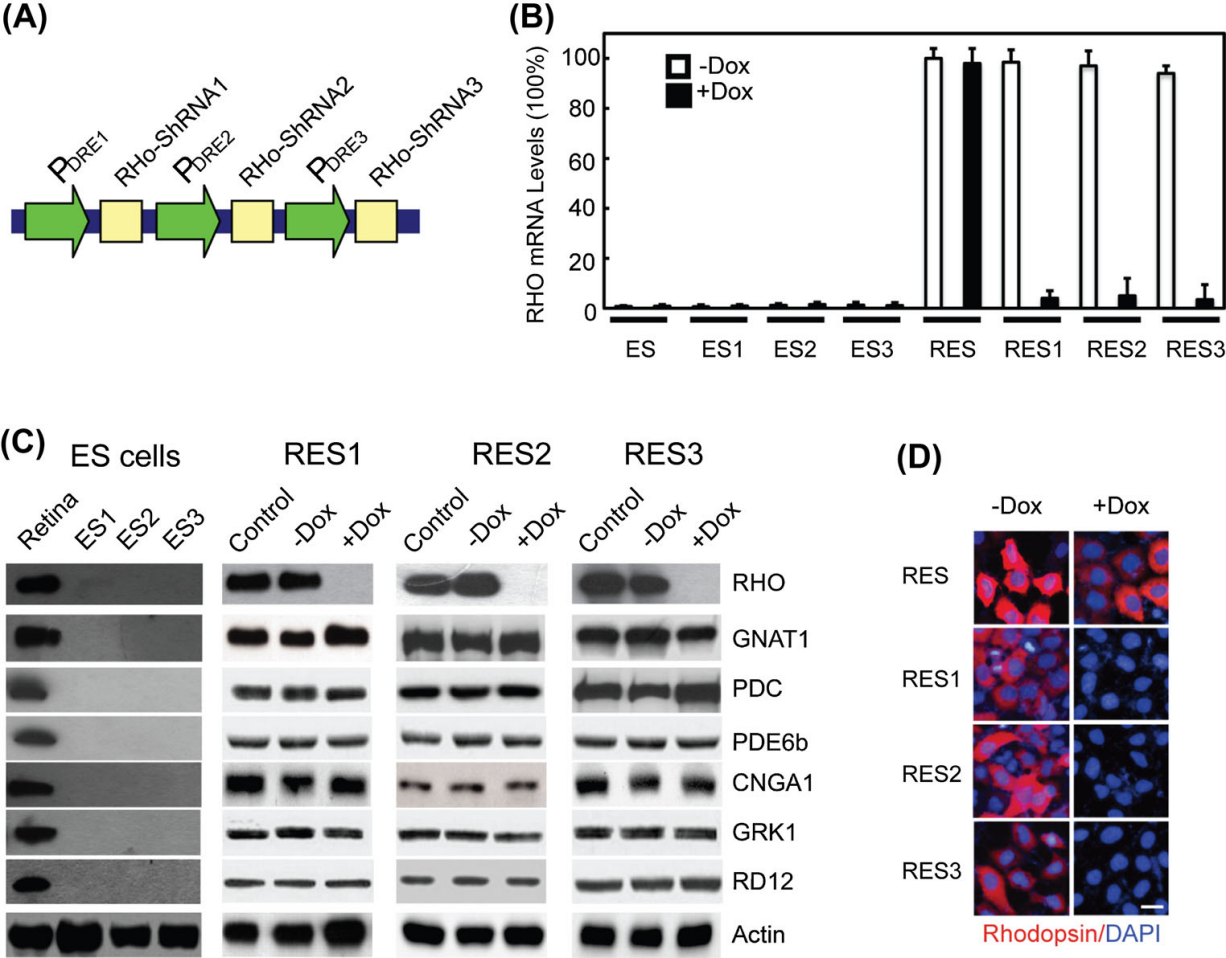
Silencing of rhodopsin expression in photoreceptor cells derived from embryonic stem cells. **a** Targeting scheme. **b** Rhodopsin mRNA levels in three clusters of cells derived from ES cells. **c** Interfered expression of the rhodopsin gene in the transgenic retina. Three G418-resistent stable ES cell lines (ES1, ES2 and ES3) carrying the rhodopsin shRNA silencing alleles and their corresponding progeny cultures (RES1, RES2 and RES3) were used in this study. WT mouse ES cells (ES) and their progenies (RES) were included as controls. Values are mean ± SEM (n = 3, *P* < 0.01); *scale bar* 10 µm. **d** Rhodopsin protein levels detected by immunofluorescence. RHO, rhodopsin; ES, embryonic stem cells; ES1–ES3, the randomly selected three G418-resistant stable ES cell lines which could express shRNA targeting Rhodopsin; RES, photoreceptor cells derived from ES; RES1–RES3, photoreceptor cells derived from ES1–ES3, respectively

To further test the general applicability of the present strategy, we also performed similar silencing experiments for cell- or tissue-specific genes in the progenies of ES cells. We targeted the murine rhodopsin gene (GenBank accession number, NM_145383) using a similar shRNA silencing cassette (Fig. 3a). Rhodopsin is a major rod photoreceptor-specific component critical for both phototransduction and outer segment (OS) disk morphogenesis in the retina (Lem et al. 1999; Palczewski 2006). We randomly selected three G418-resistant stable ES cell lines (ES1, ES2 and ES3) for further differentiation to corresponding photoreceptor cells (RES1, RES2 and RES3). qRT-PCR showed that rhodopsin mRNA levels were reduced by more than 94 % in all the photoreceptor cells differentiated from the transgenic ES cell lines after doxycycline treatment (Fig. 3b). Western-blotting assay showed that the differentiated photoreceptor cells expressed multiple retina-specific markers including RHO, GNAT1, PDC, PDE6b, CNGA1, GRK1, and RD12, while these markers were not detectable in the mES cell lines (Fig. 3c). Immunofluorescence analysis further demonstrated that the rhodopsin protein was not detectable in any of the tested cells (Fig. 3d).

### Gene silencing in transgenic animals

**Fig. 4:**
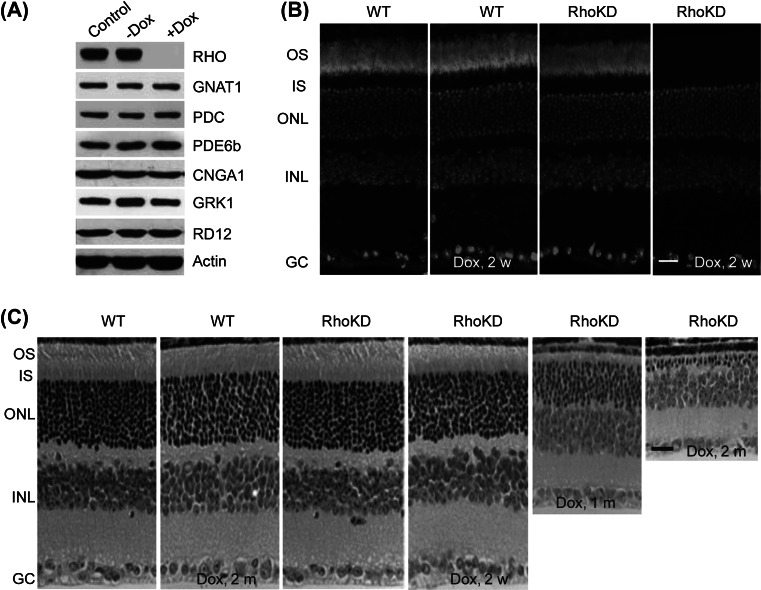
Silencing effects of the rhodopsin gene in mouse transgenic retinas. **a** Determination of levels of rhodopsin (RHO), GNAT1, PDC, PDE6b, CNGA1, GRK1, RD12, and mouse actin (m-actin) in the transgenic retina (RhoKD) by immunoblotting. Retinal proteins were extracted for immunoblotting after a 2-week doxycycline treatment. **b** Rhodopsin (*green color* in the OS layers) levels in the transgenic retinas (RhoKD) revealed by immunofluorescence. **c** Retinal morphology shown by HE staining. Mice were administered with or without doxycycline for periods indicated at the *bottom* of the corresponding panels (2 weeks, 1 and 2 months). Retinas were taken from mice at 2 months of age. Age-matched WT retinas were included as controls. *Scale bar* 20 µm. OS, outer segment; IS, inner segment; ONL, outer nuclear layer; INL, inner nuclear layer; GC, ganglion cell layer; RHO, rhodopsin; GNAT1, alpha-transducing 1; PDC, phosducin; PED6b, phosphodiesterase 6b; CNGA1, cyclic nucleotide-gated channel alpha-1; GRK1, G protein-coupled receptor kinase 1; RD12, retinal pigment epithelium 65. (Color figure online)

To investigate whether this silencing strategy was also effective in transgenic animals, we generated a rhodopsin silencing mouse model (RhoKD) by using ES1, one of the above transgenic mES cell lines. We found that the transgenic retina (RhoKD) was normally developed with a normal rhodopsin expression level and normal retinal morphology in the absence of doxycycline. On immunoblotting, the rhodopsin protein was not detectable, whereas the expression levels of multiple retina-specific markers (GNAT1, PDC, PDE6b, CNGA1, GRK1, and RD12) were minimally affected prior to obvious loss of photoreceptor cells, suggesting that the silencing effect was highly specific to the rhodopsin gene (Fig. 4a). Using immunofluorescence staining, we found that the rhodopsin protein was highly concentrated in the OS disks of the retinal rod photoreceptor cells of the transgenic mice in the absence of doxycycline. Thus, there was no substantial difference between WT and transgenic retinas in terms of rhodopsin abundance and distribution under normal conditions. However, the rhodopsin protein was not detectable after 2-week doxycycline treatment (Fig. 4a, b). As doxycycline treatment proceeded, photoreceptor degeneration became increasingly severe. Morphological analysis revealed that doxycycline did not cause abnormalities in either photoreceptor number or structure (Fig. 4c, first and second panels). The photoreceptor numbers in the transgenic retina remained comparable to those in the WT control in the absence of doxycycline (Fig. 4c, the third panel) or after the 2-week doxycycline treatment (Fig. 4c, the fourth panel), whereas the OS length was slightly shortened after the 2-week doxycycline treatment (Fig. 4c, the fourth panel). However, after 1-month doxycycline treatment, the OS length was significantly shortened even though a remarkable proportion of photoreceptor cells (>¾) remained (Fig. 4c, fifth panel). After a 2-month doxycycline treatment, less than two rows of photoreceptor cells remained in the transgenic retinas whereas the OS length was negligible (Fig. 4c, sixth panel). These morphological changes were very similar to those reported for rhodopsin knockout and dominant-negative mutant mice (Chadderton et al. 2009; Frederick et al. 2001; Li et al. 1998; Naash et al. 1993; Olsson et al. 1992).

## Discussion

Gene targeting is a powerful tool for gene function analysis and mechanistic understanding of disease conditions. However, the less than 1,000-fold lower frequency of the targeted homologous recombination event relative to random integration can necessitate screening of thousands of clones to identify a correct recombinant. To date, strategies including marker selection, promoter-trap, and viral delivery have been widely applied to boost efficiencies; however, these approaches are not always successful in achieving high-efficiency targeting.

Recently, homologous recombination-mediated disruption of the occludin gene locus in mouse spermatogonial stem cells has been shown to reach high efficiency (Kanatsu-Shinohara et al. 2006). Targeting approaches using engineered zinc-finger nucleases or TALEN technologies have also been reported to achieve biallelic disruption of mammalian genes at higher frequencies in cell lines or animals (Boch et al. 2009; Miller et al. 2011; Moscou and Bogdanove 2009; Urnov et al. 2010; Wood et al. 2011). Recently, the type II bacterial CRISPR/Cas system has been shown to be an efficient gene-targeting technology with the potential for multiplexed genome editing (Cong et al. 2013; Garneau et al. 2010; Wang et al. 2013). However, it remains to be elucidated whether these approaches are widely applicable to many other loci or species or drug-controlled targeting methods.

While the emergence of RNAi technology has led to a new era in gene targeting, most successful cases of conventional RNAi approaches are still limited to transfected cells transiently targeting the genes of interest. Our data indicated that, the silencing efficiency of an increment in both shRNA diversity, and its copy number was more effective than each individual shRNA subcassette, and the copy number increased alone shRNA construct. This observation supports the notion that different shRNA molecules might target different pathways to silence gene expression, whereas the silencing effect of each individual shRNA subcassette could be saturated. Therefore, our results suggest that an effective silencing strategy should combine a copy number increment with diverse shRNA molecules. The modified strategy proposed here presents a remarkable silencing efficiency (close to 100 % at the protein level) through synergistic interference of multiple shRNAs whose expressions are tightly controlled by engineered P_DREs_. The non-detectable levels of target proteins in all tested mammalian systems, including HEK293 cells, ES cell-derived photoreceptor cells, and the transgenic mouse model, suggest the wide applicability of our modified strategy.

One disadvantage of RNAi strategies is the potential effect of off-target silencing, while this problem can be solved by designing ShRNA silencing subcassettes matching the 5′UTR or 3′UTR of target genes in combination with phenotype reversal with their coding regions. Overall, this strategy not only achieves an ideal silencing efficiency compared to conventional RNAi approaches but also saves considerable time and labor compared to the traditional gene knockout strategy. Therefore, this synergistic shRNA silencing system may serve as a truly powerful gene targeting alternative by avoiding laborious screening of thousands of clones required by traditional knockout strategies.

## Electronic supplementary material

Below is the link to the electronic supplementary material.

Fig. S1 Silencing of SIRT1 expression in HEK293 cells by three identical shRNAs. **a** A silencing cassette for the vector harboring three SIRT1-shRNA1 subcassettes. **b** A silencing cassette for the vector harboring three SIRT1-shRNA2 subcassettes. **c** A silencing cassette for the vector harboring three SIRT1-shRNA3 subcassettes. **d** SIRT1 mRNA levels. **e** SIRT1 protein levels. Values are mean ± SEM (n = 3, P < 0.01) (TIFF 655 kb)

Supplementary material 2 (DOC 69 kb)
