# Supplementary figures and images for: Drug-inducible synergistic gene silencing with multiple small hairpin RNA molecules for gene function study in animal model
Source: Transgenic Res. 2014 Oct 1;24(2):309–17. doi: 10.1007/s11248-014-9841-9 (PMC4356887; doi:10.1007/s11248-014-9841-9)

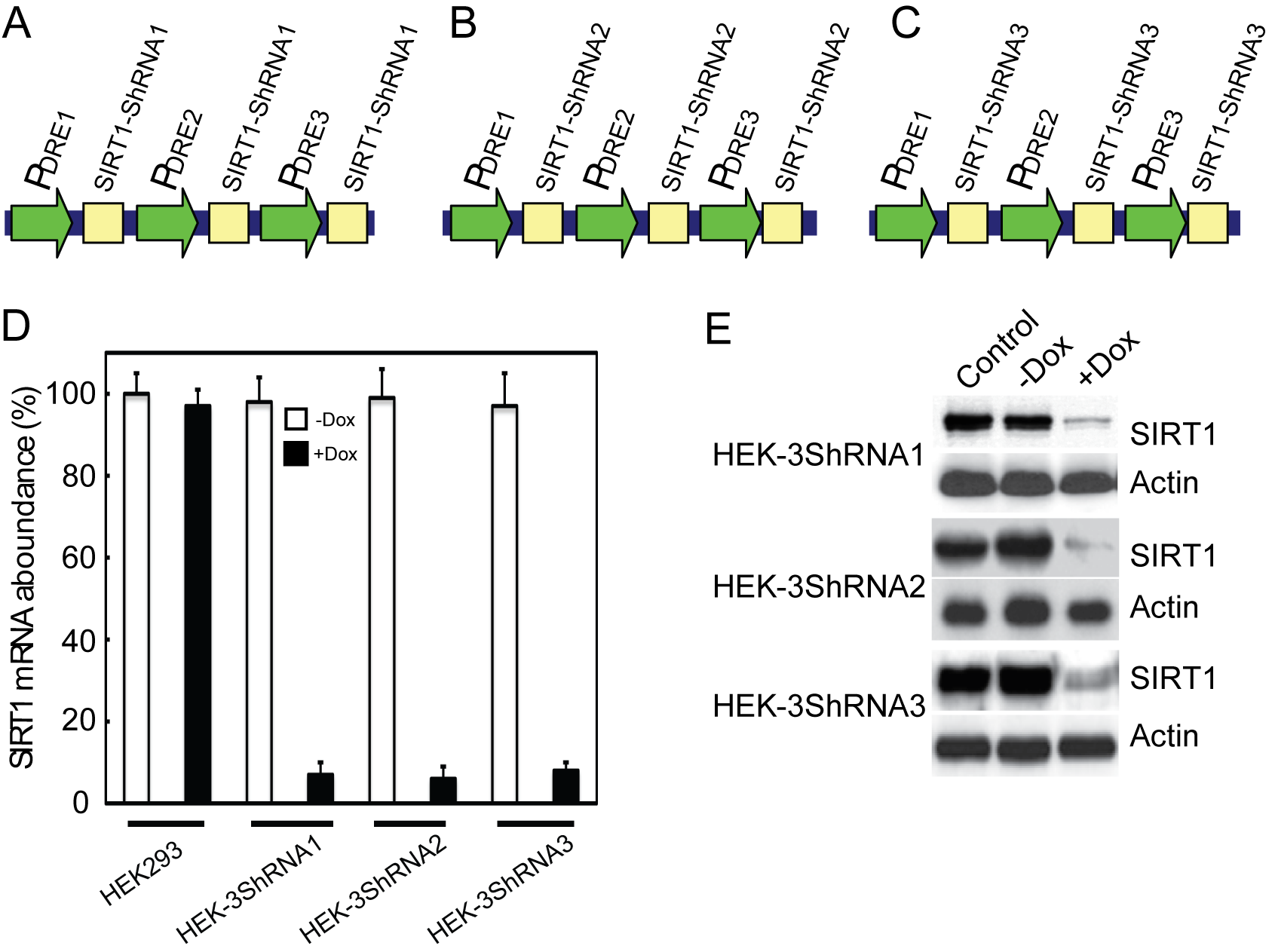

Supplement: Supplementary file 1 — Fig. S1 Silencing of SIRT1 expression in HEK293 cells by three identical shRNAs. a A silencing cassette for the vector harboring three SIRT1-shRNA1 subcassettes. b A silencing cassette for the vector harboring three SIRT1-shRNA2 subcassettes. c A silencing cassette for the vector harboring three SIRT1-shRNA3 subcassettes. d SIRT1 mRNA levels. e SIRT1 protein levels. Values are mean ± SEM (n = 3, P < 0.01) (TIFF 655 kb) [file 11248_2014_9841_MOESM1_ESM.tif]
