# Supplementary material for: Drug-inducible synergistic gene silencing with multiple small hairpin RNA molecules for gene function study in animal model
Source: Transgenic Res. 2014 Oct 1;24(2):309–17. doi: 10.1007/s11248-014-9841-9 (PMC4356887; doi:10.1007/s11248-014-9841-9)
Supplement: Supplementary file 2 — Supplementary material 2 (DOC 69 kb) [file 11248_2014_9841_MOESM2_ESM.doc]

**Supplementary Table S1. Sequences for doxycycline-responsive promoters.**

**P_DRE1_ sequence:**

5'-ctcagatctctttcgtcttcacttgagtttactccctatcagtgatagagaacgtatgtcgagtttactccctatcagtgatagagaacgatgtcgagtttactccctatcagtgatagagaacgtatgtcgagtttactccctatcagtgatagagaacgtatgtcgagtttactccctatcagtgatagagaacgtatgtcgagtttatccctatcagtgatagagaacgtatgtcgagtttactccctatcagtgatagagaacgtatgtcgaggtaggcgtgtacggtcatatgcttaccgtaacttgaaagtatttcgatttcttggctttatatatcttgtggaaaggacga

**P_DRE2_ sequence:**

5'-tttttgcccgctttccagtcgggaaacctgtcgtgccagctgcattaatgaatcggccaacgcgcggggagaggcggtttgcgtattgggcgctcttccgcttcctcgctcactgactcgctgcgctcggtcgttcggctgcggcgagcggtatcagctcactcaaaggcggtaatacggttatccacagaatcagcggccgcctcagatctctttcgtcttcacttgagtttactccctatcagtgatagagaacgtatgtcgagtttactccctatcagtgatagagaacgatgtcgagtttactccctatcagtgatagagaacgtatgtcgagtttactccctatcagtgatagagaacgtatgtcgagtttactccctatcagtgatagagaacgtatgtcgagtttatccctatcagtgatagagaacgtatgtcgagtttactccctatcagtgatagagaacgtatgtcgaggtaggcgtgtacggtcatatgcttaccgtaacttgaaagtatttcgatttcttggctttatatatcttgtggaaaggacga

**P_DRE3_ sequence:**

5'-tttttgcagattgtactgagagtgcaccatatgcggtgtgaaataccgcacagatgcgtaaggagaaaataccgcatcaggcgccattcgccattcaggctgcgcaactgttgggaagggcgatcggtgcgggcctcttcgctattacgcggtaccctcagatctctttcgtcttcacttgagtttactccctatcagtgatagagaacgtatgtcgagtttactccctatcagtgatagagaacgatgtcgagtttactccctatcagtgatagagaacgtatgtcgagtttactccctatcagtgatagagaacgtatgtcgagtttactccctatcagtgatagagaacgtatgtcgagtttatccctatcagtgatagagaacgtatgtcgagtttactccctatcagtgatagagaacgtatgtcgaggtaggcgtgtacggtcatatgcttaccgtaacttgaaagtatttcgatttcttggctttatatatcttgtggaaaggacga

The underlined sequence is the location of the core doxycycline-responsive promoter element.

**Supplementary Table S2. Oligonucleotides for the ShRNA vectors illustrated in Fig.2.**

SIRT1-ShRNA1:

SIRT1-1f: 5'-tcgaggccgacaacttgtacgacgaattcaagagattcgtcgtacaagttgtcggcttttttgagctca

SIRT1-1r: 5'-ccggtgagctcaaaaaagccgacaacttgtacgacgaatctcttgaattcgtcgtacaagttgtcggcc

SIRT1-ShRNA2:

SIRT1-2f: 5'-tcgacggcacagatcctcgaacaattttcaagagaaattgttcgaggatctgtgcctttttttctagaa

SIRT1-2r: 5'-cgcgttctagaaaaaaaggcacagatcctcgaacaatttctcttgaaaattgttcgaggatctgtgccg

SIRT1-ShRNA3:

SIRT1-3f: 5'-gggtatttatgctcgccttgctttcaagagaagcaaggcgagcataaataccttttttggatcca

SIRT1-3r: 5'-agcttggatccaaaaaaggtatttatgctcgccttgcttctcttgaaagcaaggcgagcataaataccctgca

The target gene is human sirtuin 1 (SIRT1) (GenBank accession number, NM_012238.4). The underlined sequence is the target sites of human SIRT1 mRNA.

**Supplementary Table S3. Oligonucleotides for the ShRNA vectors illustrated in Fig.S1A**

SIRT1-ShRNA1:

Sirt1-1f-1: 5'-tcgaggccgacaacttgtacgacgaattcaagagattcgtcgtacaagttgtcggcttttttgagctca

Sirt1-1r-1: 5'-ccggtgagctcaaaaaagccgacaacttgtacgacgaatctcttgaattcgtcgtacaagttgtcggcc

The target gene is human sirtuin 1 (SIRT1) (GenBank accession number, NM_012238.4). The underlined sequence is the target sites of human SIRT1 mRNA.

**Supplementary Table S4. Oligonucleotides for the ShRNA vectors illustrated in Fig.S1B**

SIRT1-ShRNA2:

SIRT1-2f-2: 5'-tcgacggcacagatcctcgaacaattttcaagagaaattgttcgaggatctgtgcctttttttctagaa

SIRT1-2r-2: 5'-cgcgttctagaaaaaaaggcacagatcctcgaacaatttctcttgaaaattgttcgaggatctgtgccg

The target gene is human sirtuin 1 (SIRT1) (GenBank accession number, NM_012238.4). The underlined sequence is the target sites of human SIRT1 mRNA.

**Supplementary Table S5. Oligonucleotides for the ShRNA vectors illustrated in Fig.S1C.**

SIRT1-ShRNA3:

SIRT1-3f-3: 5'-gggtatttatgctcgccttgctttcaagagaagcaaggcgagcataaataccttttttggatcca

SIRT1-3r-3: 5'-agcttggatccaaaaaaggtatttatgctcgccttgcttctcttgaaagcaaggcgagcataaataccctgca

The target gene is human sirtuin 1 (SIRT1) (GenBank accession number, NM_012238.4). The underlined sequence is the target sites of human SIRT1 mRNA.

**Supplementary Table S6. Oligonucleotides for the ShRNA vectors illustrated in Fig.3**

RHO-ShRNA1:

RHO-1f: 5'-tcgaggctgtaatctcgagggcttctttcaagagaagaagccctcgagattacagcttttttgagctca

RHO-1r: 5'-cggtgagctcaaaaaagctgtaatctcgagggcttcttctcttgaaagaagccctcgagattacagcc

RHO-ShRNA2:

RHO-2f: 5'-tcgacgagaatcacgctatcatgggtttcaagagaacccatgatagcgtgattctctttttttctagaa

RHO-2r: 5'-cgcgttctagaaaaaaagagaatcacgctatcatgggttctcttgaaacccatgatagcgtgattctcg

RHO-ShRNA3:

RHO-3f: 5'-ggttcgtggtccacttcaccatttcaagagaatggtgaagtggaccacgaacttttttggatcca

RHO-3r: 5'-agcttggatccaaaaaagttcgtggtccacttcaccattctcttgaaatggtgaagtggaccacgaacctgca

The target gene is murine rhodopsin (RHO) (GenBank accession number, NM_145383). The underlined sequence is the target sites of RHO mRNA.

**Supplementary Table S7. Oligonucleotides for qRT-PCR.**

SIRT1 :

SIRT1-5: 5'- cttcacccaccagggctccaact

SIRT1-3: 5'-ttcttgccgcagcagatggtggt

RHO:

RHO-5: 5'-agggaacctttgcctcatcta

RHO-3: 5'-gtggcaactctgataaatgaacc

Human Actin:

Actin-5: 5'-atgtgcgacgaagacgagacc

Actin-3: 5'-tgacccataccgaccatgacg

Mouse Actin:

mActin-5: 5'-caggcggtgctgtccctctat

mActin-3: 5'-accgataaaggaaggctggaa
